# Leaf Soluble Sugars and Free Amino Acids as Important Components of Abscisic Acid—Mediated Drought Response in Tomato

**DOI:** 10.3390/plants9091147

**Published:** 2020-09-04

**Authors:** Bojana Živanović, Sonja Milić Komić, Tomislav Tosti, Marija Vidović, Ljiljana Prokić, Sonja Veljović Jovanović

**Affiliations:** 1 Department of Life Sciences, Institute for Multidisciplinary Research, University of Belgrade, 11030 Belgrade, Serbia; bojana.zivanovic@imsi.rs (B.Ž.); sonjamilic@imsi.rs (S.M.K.); mvidovic@imgge.bg.ac.rs (M.V.); 2 Faculty of Chemistry, University of Belgrade, PO Box 51, 11001 Belgrade, Serbia; tosti@chem.bg.ac.rs; 3 Laboratory for Plant Molecular Biology, Institute of Molecular Genetics and Genetic Engineering, University of Belgrade, 11000 Belgrade, Serbia; 4 Faculty of Agriculture, University of Belgrade, 11080 Belgrade, Serbia; ljprokic@agrif.bg.ac.rs

**Keywords:** ABA, Ailsa Craig cv., arabinose, branched-chain amino acids, galactose, water deficit, *flacca* mutant, proline, re-watering

## Abstract

Water deficit has a global impact on plant growth and crop yield. Climate changes are going to increase the intensity, duration and frequency of severe droughts, particularly in southern and south-eastern Europe, elevating the water scarcity issues. We aimed to assess the contribution of endogenous abscisic acid (ABA) in the protective mechanisms against water deficit, including stomatal conductance, relative water potential and the accumulation of osmoprotectants, as well as on growth parameters. To achieve that, we used a suitable model system, ABA-deficient tomato mutant, *flacca* and its parental line. *Flacca* mutant exhibited constitutively higher levels of soluble sugars (e.g., galactose, arabinose, sorbitol) and free amino acids (AAs) compared with the wild type (WT). Water deficit provoked the strong accumulation of proline in both genotypes, and total soluble sugars only in *flacca*. Upon re-watering, these osmolytes returned to the initial levels in both genotypes. Our results indicate that *flacca* compensated higher stomatal conductance with a higher constitutive level of free sugars and AAs. Additionally, we suggest that the accumulation of AAs, particularly proline and its precursors and specific branched-chain AAs in both, glucose and sucrose in *flacca*, and sorbitol in WT, could contribute to maintaining growth rate during water deficit and recovery in both tomato genotypes.

## 1. Introduction

Drought is one of the major abiotic environmental factors that, in combination with high insolation and high temperatures, often cause detrimental losses in crop productivity [1,2,3]. In nature, plants often face a mild drought combined with other factors, thus the effect of solely water deficit is masked by the other two stresses—heat and photo-oxidation [4]. Yield productivity is affected by climate changes, which involve repeated occurrences of limited water supply combined with elevated CO_2_ and high temperature, generating enhanced plant drought tolerance [5,6]. Many recent studies are focused on plastic responses at phenotypic or gene expression levels, aiming to dissect out the genetic bases of responses to drought stress.

As a stress hormone, abscisic acid (ABA) is involved in the response to various adverse environmental factors, particularly to drought, through the regulation of specific signalling pathways and the modification of gene expression levels [7,8,9,10,11]. Under drought, ABA controls water loss through stomata closure, transpiration flux reduction, a decrease in water potential (ψ), and a reduction in leaf area [12]. Further, cell turgor is maintained through the ABA-dependent induction of osmoprotective compounds, such as amino acids (e.g., proline) and sugars [13]. The actions of ABA and reactive oxygen species (ROS) in water stress defence are interconnected. Despite the important role of particular ROS in stomatal closure and other downstream ABA signalling pathways [14], drought-induced ABA is also responsible for the induction of several antioxidative enzymes [13], crucial in protection against potential oxidative damage.

One of the main protective mechanisms against water restriction is ABA-mediated stomatal closure followed by a decrease in net photosynthetic rate and increase in photorespiration [15,16,17]. In addition, a disbalance in plant nutrients may result in nitrogen deficiency and reduced protein biosynthesis, even under mild drought [18]. Carbohydrates, besides playing an important role as osmoprotectants under drought stress, have multiple functions as signalling molecules and substrates for different cell processes [19]. Water deficit provoked the accumulation of soluble sugars (glucose and fructose) in source leaves of Arabidopsis that participated in starch biosynthesis and in sucrose transport to sink tissues [20]. While sucrose and glucose act as osmoprotectants and substrate components for cell respiration, fructose is associated with secondary metabolite synthesis [19]. The dynamics of these sugar contents under drought stress are regulated in an ABA-dependent manner [21,22].

The other common plant protective mechanism against water deficit is the accumulation of soluble sugars and other osmoprotectants, such as amino acids (e.g., proline) [23,24,25,26]. These osmotically active compounds stabilise proteins and membranes and reduce osmotic potential, aiming to prevent cellular dehydration [27]. For example, proline is involved in the stabilisation of cell membranes and various proteins and macromolecules [27,28,29]. Further, proline can act as an antioxidant and signalling molecule [30,31,32], providing the additional protection against water loss [21]. Moreover, cell wall-bound (hydroxyl)proline-rich proteins are important in maintaining the structural integrity of plant cells, particularly since the cell wall has to provide tensile strength to withstand the turgor pressure [29].

Besides acting as osmoprotectants, free amino acids (AAs) have an ameliorating role in drought-induced nitrogen uptake, through its reassimilation and maintaining protein homeostasis. Although several studies investigated the drought-related accumulation of total free AAs (as a result of proteolysis), in order to adjust the osmotic potential or to detoxify ammonia, only a few studies have been focused on the accumulation of specific AAs [33,34,35]. In Arabidopsis, the levels of numerous AAs (such as proline, glutamine, tryptophan, alanine, aspartate, ornithine, isoleucine, leucine, valine), were elevated by water deficit [33]. However, the potential specific role of particular AAs and carbohydrates during water deficit has yet to be elucidated.

Tomato is a high-value crop and one of the most widely grown vegetables [36], particularly in the Mediterranean region [37]. During the last few decades, due to climate changes, this region is strongly affected by drought during a part of the entire growing season, causing yield losses [38,39]. Thus, one of the most important requests in modern agriculture is to reveal the key metabolic pathways responsible for drought tolerance, enabling the breeders to avoid yield underperformances during water restriction [40]. Although the mechanisms underlying drought tolerance in tomato have been studied extensively, these studies were mainly focused on the effects on fruits [41,42].

The objectives of our study were: (i) to analyse the reversible effect of mild drought on primary metabolites and water status in tomato plants, with the aim to study acclimation mechanisms; (ii) to correlate the obtained results with the constitutive and drought-inducible leaf ABA content. So far, three tomato mutants (*notabilis*, *sitiens* and *flacca*) with reduced ABA content have been described [43,44,45,46]. For the third available mutant, *flacca*, according to our knowledge, no information regarding the free proteinogenic AAs and sugar profile related to its parental line was reported nor was its response to water deficit. In this work, we intended to study the sequestered effect of drought, aiming to unravel defence mechanisms directly induced by water restriction in soil.

## 2. Results

**Scheme 1:**
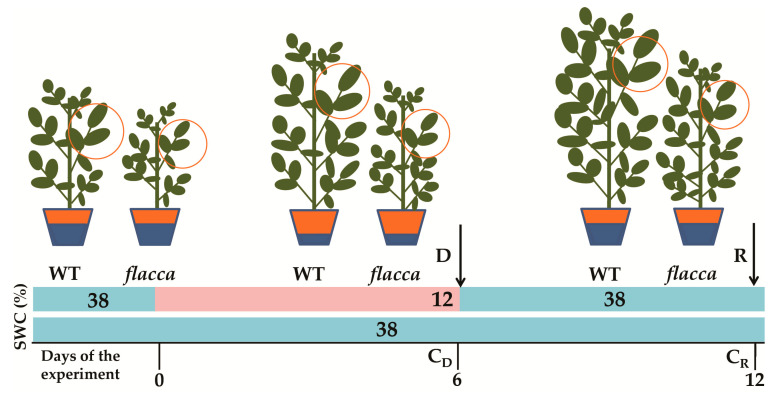
Diagram of the experimental setup. Water deficit (D) started at the beginning and lasted until the sixth day of the experiment, followed by a six day re-watering period (R), until the 12th day of the experiment. Arrows indicate harvesting points: on the sixth day (D and C_D_, respective control) and the 12th day of the experiment (R and C_R_, respective control. SWC (soil water content) was ~38% (blue line) in well-watered plants (control and re-watered plants) and reached (~12%, pink line) during the 6 day water deficit period (D). All the analyses were performed on the fourth fully developed tomato leaf (orange circle).

In order to study the influence of endogenous abscisic acid (ABA) on specific growth parameters and osmoprotectants during drought, *flacca* and wild type (WT) tomato genotypes were exposed for six days to water deficit (D), followed by six days of re-watering (R) (Scheme 1).

### 2.1. Water Deficit Effect on Leaf ABA Content, Stomatal Conductance, and Relative Water Potential

**Figure 1:**
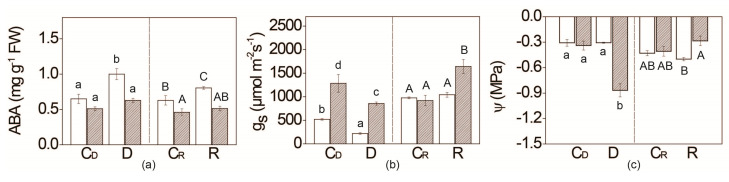
Content of ABA (**a**), values of stomatal conductance, g_s,_ (**b**) and relative water potential, ψ (**c**), in the leaves of WT (white bars) and *flacca* (patterned bars) tomato genotypes subjected to six days of water deficit (D) period followed by a six day period of re-watering (R). Respective controls corresponding to water deficit condition (C_D_) and re-watering (C_R_) are presented. Values are presented as means ±SE (*n* ≥ 4). Different letters denote significant differences between means according to Tukey HSD post hoc test *p* ≤ 0.05. Small letters represent the differences in means of drought treatment, while capital letters represent re-watering treatment.

*Flacca* mutant exhibited lower leaf ABA content in the leaves compared with WT irrespective of water supply (Figure 1, Supplementary Table S1, genotype effect *p* = 0.000110). Upon water deficit, ABA content increased by 50% in WT compared to its respective control. Six days upon re-watering, ABA level decreased to initial levels in *flacca* plants. Stomatal conductance (g_s_) in well-watered *flacca* plants on the sixth day of the experiment (C_D_, Scheme 1) was more than twice higher than in WT, while six days after (C_R_), no difference between *flacca* and WT in g_s_ was observed (Figure 1). Followed by water deficit, g_s_ was almost 40% lower than in well-watered plants in both genotypes, while upon re-watering, it significantly increased only in the *flacca* genotype.

After the six day water deficit period, *flacca* exhibited more than twice lower relative water potential (ψ) compared with WT, and also with fully irrigated plants (C_D_, Figure 1). After the re-watering period, ψ returned to values previously found in fully irrigated plants.

### 2.2. Water Deficit Effect on WT and Flacca Growth

**Figure 2:**
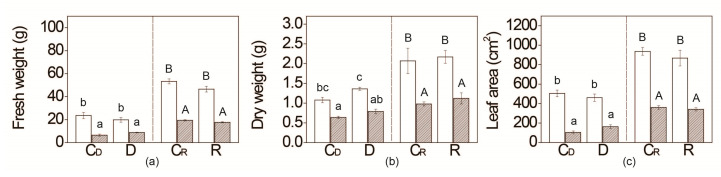
Total shoot fresh weight, FW (**a**) and dry weight, DW (**b**) per plant and leaf area (**c**) of WT (white bars) and *flacca* (patterned bars) tomato genotypes subjected to six day water deficit (D) period followed by a six day period of re-watering (R). Respective controls corresponding to water deficit condition (C_D_) and re-watering (C_R_) are presented. Values are presented as means ± SE (*n* = 4). Different letters denote significant differences between means according to Tukey HSD post hoc test *p* ≤ 0.05. Small letters represent the differences in means of drought treatment, while capital letters represent re-watering treatment.

The growth of *flacca* was slower compared with WT plants, irrespective of water supplementation, as evidenced through a lower biomass production (lower shoot fresh – FW anddry weight – DW, and leaf area), throughout the experiment (Figure 2, Supplementary Table S2). The six day water deficit period (D, 12 ± 2% soil water content; Scheme 1), had no effect on the growth rate of both WT and *flacca* as they did not differ significantly in either FW or DW. Ratios of FW and the leaf area of WT plants on the sixth and 12th day of the experiment were similar, independently of drought, while in *flacca* these ratios decreased in plants subjected to drought, compared to regularly watered *flacca* plants (Figure 2). During the period of re-watering no differences in FW and leaf area between WT plants that experienced drought and respective controls were obtained, while *flacca* plants exposed to water deficit exhibited slower growth (FW, leaf area) compared with *flacca* plants that were not subjected to water deficit (Figure 2).

### 2.3. Water Deficit Effect on Soluble Sugar Content

**Figure 3:**
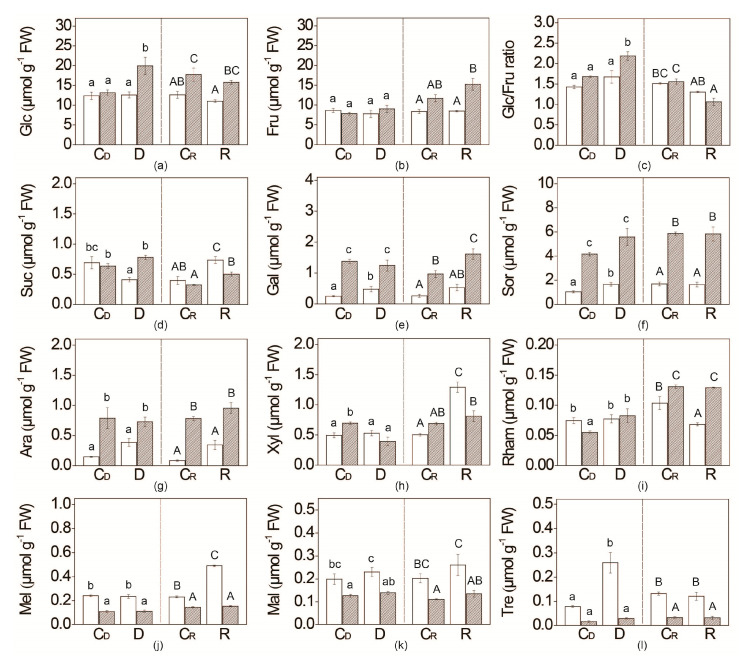
Content of soluble sugars: (**a**) glucose—Glc; (**b**) fructose—Fru; (**c**) Glc/Fru ratio; (**d**) sucrose—Suc; (**e**) galactose—Gal; (**f**) sorbitol—Sor; (**g**) arabinose—Ara; (**h**) xylose—Xyl; (**i**) rhamnose—Rham; (**j**) melibiose—Mel; (**k**) maltose—Mal; (**l**) trehalose—Tre; in the leaves of WT (white bars) and *flacca* (patterned bars) tomato genotypes subjected to six day water deficit (D) period followed by a six day period of re-watering (R). Respective controls corresponding to water-deficit condition (C_D_) and re-watering (C_R_) are presented. Values are presented as means ± SE (*n* = 4). Different letters denote significant differences between means according to Tukey HSD post hoc test *p* ≤ 0.05. Small letters represent the differences in means of drought treatment, while capital letters represent re-watering treatment.

The most abundant sugar in both tomato genotypes was glucose (11–20 µmol g^−1^ FW), followed by fructose (7–16 µmol g^−1^ FW), which together made about 80% of all measured soluble sugars (Figure 3; Supplementary Table S3). Under optimal watering conditions, the content of total soluble sugars was higher in *flacca* compared with WT plants throughout the whole experiment (Supplementary Table S4). Monosaccharides glucose and fructose was similar on the sixth day of the experiment (C_D_) between both genotypes, while it was higher in *flacca* than in WT plants on the 12th day (C_R_), as shown in Figure 3. Content of pentoses, arabinose, and xylose increased in *flacca* compared to WT in both time points, while rhamnose content was higher in WT on the sixth day of the experiment (C_D_). Under optimal water supply (C_D_), the contents of disaccharides, trehalose, melibiose and maltose, were lower in *flacca* in comparison to WT, while sucrose level was similar in both genotypes in control conditions (Supplementary Tables S3 and S4).

The total content of measured soluble sugars remained unchanged in WT plants subjected to water deficit, while in *flacca* it increased by 30% compared with fully irrigated plants (Supplementary Table S3). Further re-watering did not influence the content of total sugars in both genotypes. Upon six days of water deficit, contents of galactose, sorbitol, arabinose, and trehalose increased in WT plants (Figure 3). On the other hand, *flacca* plants accumulated more glucose and sucrose upon water restriction compared to the optimal watering regime. The glucose/fructose ratio upon water deficit was higher in *flacca*, while upon re-watering it decreased, reaching approximately the value of 1. Drought-responsive sugars in *flacca*: galactose and sorbitol, as well as arabinose, were constitutively more abundant in *flacca*, while di- and oligosaccharides (maltose, melibiose, raffinose, and trehalose) were more abundant in WT than in *flacca*, irrespective of water supply (Figure 3, Supplementary Tables S3, S4). Except for trehalose, the amount of these three oligosaccharides did not change significantly in both genotypes upon water deficit.

### 2.4. Water Deficit Effect on Free Amino Acids Content

**Figure 4:**
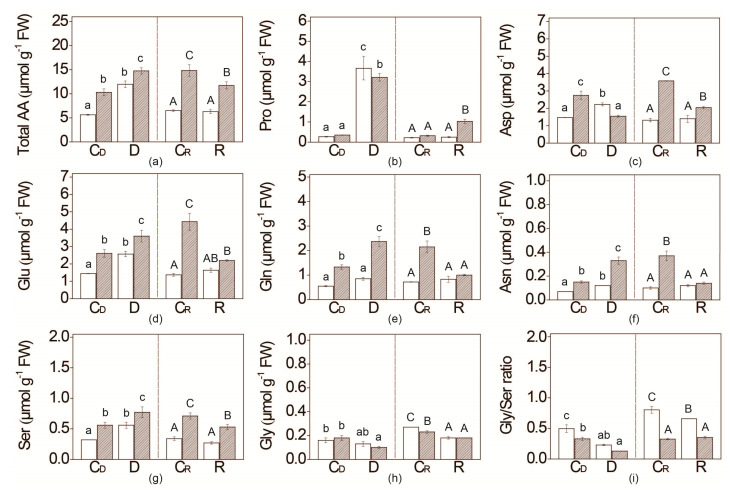
Content of free amino acids (AAs): (**a**) total AAs; (**b**) Proline—Pro; (**c**) aspartate—Asp; (**d**) glutamate—Glu; (**e**) glutamine—Gln; (**f**) Asparagine—Asn; (**g**) serine—Ser; (**h**) glycine—Gly; (**i**) Gly/Ser ratio; in the leaves of WT (white bars) and *flacca* (patterned bars) genotypes subjected to six day water deficit (D) period followed by a six day period of re-watering (R). Respective controls corresponding to water deficit condition (C_D_) and re-watering (C_R_) are presented. Values are presented as means ±SE (*n* = 4). Different letters denote significant differences between means according to Tukey HSD post hoc test *p* ≤ 0.05. Small letters represent the differences in means of drought treatment, while capital letters represent re-watering treatment.

**Figure 5:**
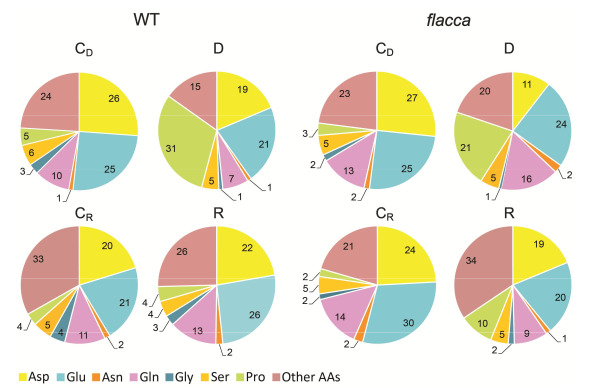
Distribution of free amino acids (AAs) (%) in the leaves of WT and *flacca* tomato genotypes subjected to six day water deficit (D) and six day re-watering (R) period. Respective controls corresponding to water-deficit condition (C_D_) and re-watering (C_R_) are presented. Values are presented as means and standard errors were below 20% (*n* = 4). The abbreviations are explained in Figure 4.

Total free amino acids (AAs) content, as well as the individual amino acids of both genotypes, fluctuate during development. Nonetheless, the total free AAs content was approximately two times higher in *flacca* compared to WT plants grown irrespective of the watering regime (Figure 4, Supplementary Tables S5, S6). The most abundant AAs in both tomato genotypes were aspartate, glutamate and glutamine, and their contribution was similar between genotypes (Figure 5).

The most striking effect of water deficit on particular AA was the significant increase in proline content by almost 10 times in both genotypes (Figure 4). After re-watering, the content of proline dropped to the control levels (C_R_) in WT; however, it even increased in *flacca,* reaching a 3-times higher level compared with the respective control. Additionally, proline content decline slower in *flacca* compared with WT, and remain at constant level (increased than in control plants) over 12 days after re-watering started (data not shown). In *flacca*, water deficit induced a decrease in aspartate amount simultaneously with an increase in glutamate. On the other hand, in WT aspartate and glutamate were significantly increased upon water restriction.

Upon re-watering (R), contents of aspartate, glutamate, glutamine and asparagine in the leaves of WT decreased to the same amount as in plants that were not subjected to water deficit (C_R_), while in *flacca* these amounts dropped below C_R_ values.

The response of AAs directly linked with photorespiration—i.e., serine and glycine, to water deficit was opposing in *flacca* plants. The amount of glycine decreased after a six day water deficit period in *flacca* plants, resulting in a twice lower glycine/serine ratio compared with the respective control plants. In WT plants, glycine/serine decreased twofold too, but only due to an increase in the amount of serine upon water deficit (Figure 4).

Furthermore, the percentage of specific AAs in their total pool changed as a consequence of different water supplementation regimes (Figure 5). Water deficit induced a significant increase in proline ratio (from 3–5% in both genotypes to 31% in WT and 21% in *flacca*) and decrease in aspartate ratio (from 26–27% in both genotypes to 19% in WT to 11% in *flacca*). Upon re-watering the contribution of aspartate in the total pool of free AAs in *flacca* increased again compared to the water deficit, while ratio of glutamate decreased (Figure 5).

## 3. Discussion

### 3.1. Genotype Flacca Is a Suitable Model System for Studying Tomato Response to Water Deficit

*Flacca* genotype has been characterised as a tomato mutant exhibiting lower abscisic acid (ABA) content (by 20–50% related to its respective parental line) in the shoot [44,47,48]. Accordingly, in our studies, *flacca* genotype grown under optimal conditions, exhibited 20–30% decreased leaf ABA content compared with its wild type (WT), Ailsa Craig cv.

ABA is most often considered as a plant growth inhibitor [47]. In our study, lower ABA level in *flacca* resulted in retarded leaf and stem growth, similarly as observed in other studies [47,49,50,51]. Moreover, in another ABA-deficient tomato mutant, *sitiens,* the relative growth rate was 22% lower in comparison with respective WT [46]. Further, reduced leaf area, shoot length and total plant dry weight was observed in the third described ABA-deficient tomato mutant, *notabilis*, compared with its parental line [45].

It is known that ABA is a key signal in the regulation of stomatal aperture and resistance [52]. Therefore, it was not surprising that, in *flacca*, stomata exhibited higher conductance (Figure 1). Besides *flacca*, similar results were observed for *sitiens*, and *notabilis*, leading to higher transpiration and CO_2_ assimilation rates [45,46,47,50]. According to these results, one could expect higher biomass production in ABA-deficient mutants—i.e., *flacca*. However, it was shown that growth inhibition in *flacca* is preferably caused by the elevated production of ethylene, plant growth inhibitor, and its predominant action [12]. Taken together, these results suggest that the ABA threshold necessary for normal growth and other phenotypic traits is much higher than that usually reported for ABA deficient genotypes. Our results indicate that a lesser ABA decrease in *flacca* compared to WT is enough to trigger mechanisms related to the fitness of these mutant plants. Therefore, it is required to reveal the precise regulation of cellular ABA homeostasis to maintain normal growth and physiology.

Increased g_s_, as a result of lower ABA levels, could induce more water loss in *flacca,* even under optimal water regime; however, no difference in relative water potential (ψ) was detected in WT plants (Figure 1). Similar effects of the ABA-mediated increased stomatal conductance (g_s_) on the relative water content were observed in the case of *sitiens* mutant [46]. This result may emphasize the contribution of alternative compensating strategies in our *flacca* plants. Common protective strategy against cellular dehydration is elevated accumulation (millimolar concentrations) of reduced forms of water-soluble sugars or sugar alcohols, serving as osmoprotectants [23]. Indeed, 2–4 times higher constitutive levels of soluble sugars, particularly sorbitol, galactose and arabinose were observed in *flacca* compared with WT plants (Supplementary Table S3; Figure 3). In addition, the amounts of fructose in well-watered *notabilis* were higher than in WT plants [36]. On the contrary, in *sitiens* genotype, the level of total sugars was almost 20% lower than in WT [46]. Besides sugars, free amino acids (AAs) particularly proline, have been proved to serve as osmoprotectants in various plant species under drought, cold shock and salt stress [25], while tobacco mutants with higher levels of proline exhibited enhanced osmotolerance [53]. Similarly, as found in our study in *flacca*, Ntatsi et al. [45] have reported higher AAs level in *notabilis* mutant compared with its WT. Thus, we suggest that accumulated proline and soluble sugars in *flacca* contributed to maintaining the optimal ψ, despite lower ABA level and increased g_s_. With a higher level of osmoprotectants in *flacca* than in WT under optimal water conditions, we intended to use *flacca* as a suitable model system for studying the influence of lower endogenous ABA content on drought-adaptation strategies.

### 3.2. Water Deficit Had No Effect on Flacca Growth, Although It Provoked Relative Water Potential Drop

Expectedly, water deficit induced an increase in ABA content in WT tomato plants, leading to stomatal closure and keeping the optimal ψ (Figure 1). Oppositely, although g_s_ in *flacca* declined by 40%, ψ drastically decreased upon water restriction, keeping the ABA content unchanged. Thus, the g_s_ reduction in *flacca* can be explained with altered pH dependency and by the involvement of the alternative hydraulic regulative mechanism of stomata opening [54,55], opposite to the dominant ABA-dependant pathway revealed in WT [11]. Nevertheless, the *flacca* genotype possesses an efficient mechanism to cope with low ψ, as evidenced by no effect on total shoot FW and DW (Figure 2). Therefore, we suggested an additional biochemical mechanism that caused *flacca* to preserve growth rate under well-watered conditions.

### 3.3. Water Deficit Stimulated Accumulation of Specific Sugars

The objective of our work was to reveal which soluble sugar and proteinogenic AAs specifically are affected by water deficit concerning the endogenous ABA level, and to correlate this observation with their possible roles in defence against water restriction by acting as osmolytes, precursors for energy-associated metabolites, ROS scavengers, as well as potential regulatory and signalling molecules.

In WT plants, sorbitol, trehalose, galactose, and arabinose doubled upon water restriction (Figure 3). All these sugars are considered as effective osmolytes and their accumulation has been previously reported in rice plants subjected to salt stress [56]. Moreover, trehalose, via trehalose-6-phosphate, regulates starch breakdown The increased glucose/fructose ratio in WT upon water deficit might be regarded as the indication for starch degradation in WT [57,58]. Indeed, sucrose content in the mature fourth leaf was decreased upon water deficit, suggesting its possible transfer to young leaves under stress conditions. Under drought and ABA treatment, sucrose export from source to sink tissues increases [20,22]. In this way, the accumulation of sucrose in sink tissues increases, resulting in a decreased hexose-to-sucrose ratio. In addition, drought stress increases the expression and the activities of acid invertase enzymes in the leaves, leading to irreversible hydrolysis into glucose and fructose [59,60]. 

Galactose accumulation during drought in our WT plants was also observed in apple leaves [61] and potato leaves and this is consistent with the assumed role of galactose in alleviating osmotic stress [62]. Furthermore, arabinose, galactose, and mannose, as well as xylose, are important components in plant cell wall construction, as a part of different O- and *N*-glycoproteins involved in plant response to different environmental factors [63,64]. Thus, galactose and arabinose might contribute to cell wall stiffening in WT during water restriction, as evidenced with unchanged dry weight in comparison to control plants.

In *flacca*, but not in WT plants, water deficit induced an increase in total soluble sugars. Accordingly, in drought susceptible rice, the variety level of total soluble sugar increased under the water deficit, while in drought-resistant cultivar, no changes were detected [65]. The increase in glucose in *flacca* plants exposed to water deficit can be linked with the increase in glucose/fructose ratio and maltotriose (Supplementary Table S3, Figure 3), additionally implying starch degradation followed by sugar reallocation. Obtained glucose could serve as a building block for other osmoprotectants—e.g., sorbitol that was 3-times higher than in WT plants. Similarly, as a part of UV-B tolerance strategy, the stimulation of starch and sucrose breakdown, followed by carbon allocation in the form of hexoses and pentoses, provided the building blocks for biosynthesis of UV-B protective compounds [58]. Moreover, starch degradation was also observed in *sitiens* mutant exposed to salt stress [46]. The accumulation of soluble sugars, such as sucrose, glucose and fructose, in *flacca* source leaf was the result of decreased transport of photosynthates to the sink tissues by ABA-mediated mechanisms of growth inhibition (Figure 2) [60]. To unravel the mechanism of source-sink transportation of sucrose in *flacca*, future work on carbon allocation in the whole plant, as well as the expression level of sucrose transporters is required.

Soluble sugar content was restored to control values in both genotypes after re-watering, which is correlated to a previous study done by Wu et al. [66]. Likewise, the content of glucose and fructose in recovered plants was re-established, similarly as in the case of two genotypes of tedera and lucerne leguminous after rehydration in the study of Foster and co-workers [67].

### 3.4. Water Deficit Provoked Accumulation of Specific Amino Acids

Besides soluble sugar characterisation, we analysed changes in AA profile in both tomato genotypes induced by water deprivation. Both tomato genotypes accumulated large amounts of AAs upon water deficit (Figure 4; Supplementary Table S5). This increase is consistent with previous reports in drought-tolerant sesame [35], rice [68], maize [69] and barley [70]. The elevation in the AA pool can be correlated with the enhanced proteolysis, contributing to osmotic adjustment under water-stress conditions [34]. However, in our study, no difference in leaf protein content per g of dry weight upon water restriction was observed irrespective of genotype (data not shown).

The most striking change in AAs profile induced by water deficit was related to 9-fold increased proline accumulation in both tomato genotypes. In addition, glutamate, the main precursor in stress-induced proline biosynthesis [32], was strongly accumulated in both genotypes subjected to water deficit. The drought-induced accumulation of proline in leaves has been well documented for numerous species [25]. Besides acting as an osmoprotectant, proline has several functions: stabilisation of peptide backbone and prevention of protein aggregation, common under cellular dehydration, cell wall fortification via (hydroxyl) proline-rich proteins and the scavenging of the drought-induced ROS [29,71,72]. Moreover, in *sitiens* mutant, proline content has been tripled upon exposure to salt stress, but not in WT [46].

One of the mechanisms for drought-induced proline biosynthesis is dependent on ABA [71,73,74]. In our study, proline accumulation followed a similar trend as ABA content increased in both WT and *flacca* plants subjected to water deficit, resulting in slightly lower values than in WT (Figure 1 and Figure 4). The steep decrease in proline content after the six day re-watering period might be considered as a contribution to cell wall stiffening, demonstrating high proline significance in tomato acclimation to water deficit. Additionally, only in re-watered *flacca* plants, proline content retained a higher level compared with controls. Very recently, Lukic et al. [75] reported that significantly increased levels of antioxidative enzymes under drought remained elevated over weeks could be linked with better performances in plants subjected to upcoming stress. Proline accumulation as a part of stress memory could help drought-sensitive *flacca* mutants (reduced ψ, Figure 2) to better cope with repeated water deficit. Future research should reveal whether proline content in these two genotypes is involved in short-term drought stress memory, preparing *flacca* plants for enhanced response to upcoming drought.

Our results also showed that the mechanism involved in proline biosynthesis under drought stress was not ABA-dependent or that ABA content in *flacca* was under the ABA threshold required for proline biosynthesis activation. Besides its role as osmoprotectant [32,76], proline is considered as an important constituent of the cell wall matrix [77,78]. We propose here that leaves of *flacca* might be thinner partially due to the impaired reallocation of proline to extracellular space and biosynthesis of cell wall proteins, such as hydroxyproline-rich glycoproteins or (hydroxyl)proline-rich proteins, which was implicated by a delayed decrease in proline during re-watering (Figure 4).

The metabolic pathways involving aspartate, asparagine, threonine, alanine, ornithine and some branched-chain AAs (BCAAs) like valine and leucine were related to water deficit tolerance in tomato plants with unaffected ABA content. For example, the accumulation of BCAAs was more likely dependent on ABA content since it was omitted in *flacca* plants. It was proposed that BCAAs play an important role in plant drought tolerance as an alternative source of respiratory substrates [33]. Besides, the degradation of BCAAs donates electrons in the respiratory chain under stress condition [79]. Some of BCAAs degradation products during drought are substrates for tricarboxylic acid cycles, so its presumed role might be providing the alternative carbon sources for plants during stress. Numerous papers reported levels of some BCAAs were elevated during drought [80,81,82] and very fast after rehydration returned to control values which indicates that BCAAs breakdown is closely related to external conditions [83]. Urano et al. [21] has shown that the enzyme involved in the final biosynthesis step of BCAAs (BCAT) was induced in Arabidopsis WT in drought, while it was quite diminished in *NCED3* gene (*nc3-2*) knockout mutants, which implies ABA has a role in the expression of BCAT and consequently the regulation of BCAAs accumulation. Due to different changes in leucine, isoleucine and valine content in WT and *flacca* during water deficit, we may speculate that not only ABA contributes to BCAAs homeostasis.

We can assume that, during water deficit, WT tomato plants exhibited increased photorespiration arising from decreased stomatal conductance in response to dehydration-induced ABA. Similar findings related to high photorespiration induced by drought and stomatal closure were reported [84]. Indeed, the glycine/serine ratio strongly correlated with photorespiratory flux [85], was lower in both genotypes upon water deficit and increased by 2–3 times upon re-watering. Moreover, as ammonia released from glycine in photorespiration is transferred back to glycine via serine, alanine or aspartate, the negative correlation of alanine and aspartate with photorespiratory flux was suggested, too [85]. However, in our study, both alanine and aspartate contents were increased in WT upon water deficit, indicating serine as a preferential NH_3_ donor during drought. In addition, a significant accumulation of glutamine was observed in *flacca* subjected to water deficit, which could be related to the re-assimilation of drought-induced ammonia accumulation, as suggested in the leaves of drought-sensitive and drought-tolerant mulberry cultivars exposed to water deficit [34].

Free asparagine acts as nitrogen storage and nitrogen transport form in many plant species due to its high N/C ratio. The asparagine/aspartate ratio has been considered as a marker of nitrogen status in plants [86]. In WT tomato plants, asparagine/aspartate ratio remained unchanged upon water deficit, while it increased almost four times in *flacca* plants (Figure 4), suggesting better nitrogen supplementation, possible via alternative mechanisms.

## 4. Materials and Methods

### 4.1. Plant Material and Experimental Conditions

Seeds of tomato (*Lycopersicon esculentum* Mill. cv. Ailsa Craig.), wild type (WT) and its ABA-deficient mutant *flacca*, were germinated in the commercial substrate (Klasmann Potground H). Seedlings were grown up to the phase of four developed leaves in small pots and after that period transferred to larger pots (a depth of 24 cm, with approximately 800 g of the same substrate). Plants were grown under controlled conditions (14 h photoperiod, 200 μmol m^−2^ s^−1^, 50% relative humidity and day/night temperature of 26 °C/18 °C) and were irrigated daily to maintain a volumetric soil water content (SWC) of 38 ± 2% as shown in Scheme 1, measured by ML3 Theta Probe Soil Moisture sensor (Delta-T Device, Ltd., Cambridge, UK). Six days after transfer, in the phase of six fully developed leaves, half of all plants were subjected to water deficit treatment (D) by water withdrawal for six days and the rest of the plants were regularly watered throughout whole experiment (control plants: C_D_ and C_R_). After achieving 12 ± 2% of SWC, half of the plants were harvested (D) together with their respective control (C_D_), while the other half was fully irrigated and re-watered (R) during the next six days (Scheme 1). Plants previously exposed to water deficit reobtained initial SWC (38 ± 2%) already after the first day of the re-watering phase. On the 12th day of the experiment (after the six day re-watering period) plants were harvested (R), together with their respective controls (C_R_), as shown in Scheme 1. For each treatment, four biological replicates of both genotypes were prepared. All the analyses were performed on the fourth fully developed tomato leaf (Scheme 1).

### 4.2. Morphological and Physiological Parameters

Above-ground biomass of both tomato genotypes was determined upon six day water deficit period and followed by a six day re-watering period and expressed as a fresh weight (FW) of shoots, while after drying (at 70 °C for 72 h), their dry weight (DW) was measured. Total leaf area of both genotypes was measured using LI-3100 areameter (LI-COR, Lincoln, NE, USA). Measurements were done with four different plants per genotype and treatment.

Stomatal conductance (g_s_) was measured by AP4 Leaf Porometer (Delta-T Devices, Ltd., Cambridge, UK) and relative water potential (ψ) was measured by a pressure chamber (Soil Moisture Equipment Corp., Santa Barbara, CA, USA). Both g_s_ and ψ of differently watered tomato genotypes were determined on the 6th and 12th day of the experiment, with four different plants per genotype used.

### 4.3. Biochemical Analysis

For determination of abscisic acid (ABA), soluble sugars, and free amino acids (AAs) the fourth fully developed tomato leaf (from four different plants per genotype and per treatment) was harvested in the growth chamber, 5 h upon light on, immediately frozen in liquid nitrogen and stored at −80 °C for further analysis.

#### 4.3.1. Extraction and Analysis of ABA

Determination of ABA content in the leaves of tomato plants was performed by indirect enzyme-linked immunosorbent assay (ELISA) according to Asch [87] and Quarrie et al. [88]. 

Frozen leaf tissues were homogenised in liquid nitrogen, extracted in ultrapure water (1/10, *w/v*) with 1% polyvinylpyrrolidone (PVP), shortly exposed to high temperature in the water bath and transferred to over-night shaking at 4 °C. Homogenate was centrifuged at 14,000 *g* for 15 min at 4 °C and obtained supernatants were diluted 7 times ultrapure water for further analyses. ABA concentration was determined by the ELISA method using a MAC 252 monoclonal antibody for ABA (John Innes Centre, Colney, Norwich, UK). Measurements were performed at 405 nm by microplate reader (Nunc: F96 MaxiSorp).

#### 4.3.2. Extraction and Analysis of Soluble Sugars

Content of soluble carbohydrates was measured according to Vidović et al. [58]. Briefly, approximately 0.1 g of frozen leaf tissue was homogenized in liquid nitrogen, extracted in 1 mL of ultrapure water (1/10, *w/v*) and placed in an ultrasonic bath at room temperature for 30 min. Samples were centrifuged at 14,000 *g* for 10 min at 4 °C. The pellet was re-extracted with ultrapure methanol in the same manner as previously described with water. Obtained supernatants were pooled together for further analysis.

Chromatographic separations were performed using a DIONEX ICS 3000 DP liquid chromatography system (Dionex, Sunnyvale, CA, USA) equipped with a quaternary gradient pump (Dionex, Sunnyvale, CA, USA). The carbohydrates were separated on a CarboPac^®^ PA100 pellicular anion–exchange column (4 × 250 mm) (Dionex, Sunnyvale, CA, USA) at 30 °C. The mobile phase consisted of the following linear gradients (flow rate, 0.7 mL min^−1^): 0–5 min,15% A, 85% C; 5.0–5.1 min,15% A, 2% B, 83% C; 5.1–12.0 min, 15% A, 2% B, 83% C; 12.1 min, 15% A, 4% B, 81% C; 12.1–20.0 min 15%A, 4% B, 81% C; 20.0–20.1 min 20% A, 20% B 60% C; 20.1–30.0 min 20% A, 20% B 60% C; where A was 600 mM sodium hydroxide, B was 500 mM sodium acetate and C was ultrapure water. Before the analyses, the system was preconditioned with 15% A and 85% C for 15 min. The injection volume was 25 µL. The electrochemical detector consisted of gold as the working and Ag/AgCl as the reference electrode [89].

#### 4.3.3. Extraction and Analysis of Amino Acids

Amino acids were determined following a modified protocol by Noctor and Foyer [90]. Leaf material was ground in liquid nitrogen and free AAs were extracted in 50% methanol (1:10, *w/v*), followed by centrifugation for 10 min at 4 °C at 10,000 *g*. The aliquots of the obtained supernatants were diluted 10 times and mixed with an equal volume of 0.5 M sodium borate buffer (pH 9.5), 0.4 M *ortho*-propionic acid (OPA) and β-mercaptoethanol (9:1:0.2, *v:v:v*) for derivatisation.

Free AAs were identified and quantified by HPLC-PDA (LC-20AB Prominence Liquid Chromatograph, Shimadzu, Kyoto, Japan) with fluorescence detection. The excitation wavelength was set up at 340 nm, and the emission at 455 nm. The elution gradient was formed with buffer A: 20 mM sodium phosphate buffer pH 6.8: methanol: THF (90:9:1, *v:v:v*) and buffer B (20 mM sodium phosphate buffer pH 6.8: methanol: THF, 40:59:1). The following elution gradient was applied: 0–5 min A = 100%, B = 0%; 5–30 min A = 70%, B = 30%; 30–35 min A = 40%, B = 60%; 35–45 min A = 50%, B = 50%; 45–45 min A = 30%, B = 70%; 45–70 min A = 0%, B = 100%; 70–80 min B = 100%; 80–90 min B = 100%. Injection volume was 20 µL.

Since the fluorescent HPLC assay used for the bulk of amino acids was not applicable for proline determination, it was measured spectrophotometrically according to Bates et al. [91] with minor modifications. Frozen leaf tissues were homogenised in liquid nitrogen, extracted in 3% sulfosalicylic acid (1:10, *w:v*) and centrifuged at 14,000 *g* for 10 min at 4 °C. The supernatant was mixed with the same volumes of acidic ninhydrin and glacial acetic acid and incubated for 60 min at 100 °C. The reaction was stopped by placing samples in ice-bath, followed by the addition of toluene (1:1, *v:v*) and the upper organic phase was used for proline determination. The proline concentration was calculated based on absorbance at 520 nm using a standard curve.

### 4.4. Statistical Analysis

Significant differences in g_s_ and ψ values, morphological parameters, and ABA, sugar and amino acid contents between different water regimes and genotypes were determined using a two-way ANOVA test. The significance threshold value was set at *P* ≤ 0.05. Following two-way ANOVA analysis, the Tukey post hoc test was used for specific comparisons among experimental groups (*P* ≤ 0.05). The experimental data were analysed using software package Statistica 8.0.

## 5. Conclusions

Tomato mutant *flacca* provided strong evidence that ABA threshold necessary for normal growth, g_s_ and non-wilting phenotype is much higher than usually reported for ABA deficient genotypes. On the contrary, the accumulation of specific amino acid proline under water deficit was irrespective of lower ABA content, suggesting the involvement of other mechanisms or lower ABA threshold required for triggering drought-induced proline accumulation.

We showed that the *flacca* mutant exhibited constitutively higher levels of soluble sugars and free AAs compared with WT that could prevent possible cellular dehydration caused by higher stomatal conductance, due to lower ABA content. In addition, this is the first report of an extensive profile of proteinogenic AAs constitutively exhibited in *flacca*.

In summary, we suggest that the accumulation of AAs, particularly proline and its precursors and specific branched-chain AAs in both genotypes, and glucose and sucrose in *flacca*, and sorbitol in WT, could have an important role in maintaining growth rate during water deficit and recovery. Considering the significant accumulation of osmoprotectants under drought, further research should explore the specific function of particular AAs and sugars in different metabolic pathways, such as photorespiration, TCA cycle, antioxidative defence, as well as potential regulatory and signalling mechanisms that should improve crop tolerance to drought and reduce yield losses.

## Supplementary Materials

The following are available online at https://www.mdpi.com/2223-7747/9/9/1147/s1. Table S1: Statistical analysis of the results (two-way ANOVA) to test the influence of the treatment (drought—D or recovery—R) and two genotypes (WT and flacca) and their interactions on the amount of ABA, stomatal conductance and relative water potential in tomato leaves. Table S2: Statistical analysis of the results (two-way ANOVA) to test the influence of the treatment (drought—D or recovery—R) and two genotypes (WT and flacca) and their interactions on the fresh and dry weight and the leaf area in tomato leaves. Table S3: Content of soluble sugars in the leaves of WT and flacca tomato genotypes subjected to six day water deficit (D) period followed by a six day period of re-watering (R). Table S4: Statistical analysis of the results (two-way ANOVA) to test the influence of the treatment (drought—D or recovery—R) and two genotypes (WT and flacca) and their interactions on the soluble sugars content. Table S5: Content of free amino acids (AAs) (µmol g−1 FW) in the leaves of WT and flacca tomato genotypes subjected to six day water deficit (D) period followed by six day period of re-watering (R). Table S6: Statistical analysis of the results (two-way ANOVA) to test the influence of the treatment (drought—D or recovery—R) and two genotypes (WT and flacca) and their interactions on the free amino acids content.
