# Supplementary material for: Leaf Soluble Sugars and Free Amino Acids as Important Components of Abscisic Acid—Mediated Drought Response in Tomato
Source: Plants (Basel). 2020 Sep 4;9(9):1147. doi: 10.3390/plants9091147 (PMC7570426; doi:10.3390/plants9091147)
Supplement: Supplementary file 1 [file plants-09-01147-s001.pdf]

Supplementary Table S1. Statistical analysis of the results (two-way ANOVA) to test the influence of the treatment (drought – D or rewatering – R) and two genotypes (WT and *flacca*) and their interactions on the amount of ABA, stomatal conductance and relative water potential in tomato leaves.

Trait	Source of variation	df	F	*p*	Trait	Source of variation	df	F	*p*
**ABA (D)**	Genotype	1	21.31	0.000110	**ABA (R)**	Genotype	1	41.65	0.000003
	Treatment	1	20.37	0.000143		Treatment	1	10.13	0.004671
	Genotype x Treatment	1	2.53	0.125117		Genotype x Treatment	1	2.54	0.126837
**g_s (D)**	Genotype	1	238.13	0.000000	**g_s (R)**	Genotype	1	5.88	0.027575
	Treatment	1	72.62	0.000000		Treatment	1	16.61	0.000882
	Genotype x Treatment	1	13.99	0.000638		Genotype x Treatment	1	11.18	0.004127
**ψ(D)**	Genotype	1	46.30	0.000004	**ψ(R)**	Genotype	1	7.30	0.019243
	Treatment	1	35.80	0.000019		Treatment	1	0.53	0.481897
	Genotype x Treatment	1	34.24	0.000025		Genotype x Treatment	1	4.55	0.054340

Supplementary Table S2. Statistical analysis of the results (two-way ANOVA) to test the influence of the treatment (drought – D or re-watering – R) and two genotypes (WT and *flacca*) and their interactions on the fresh and dry weight and leaf area in tomato leaves.

Trait	Source of variation	df	F	*p*	Trait	Source of variation	df	F	*p*
**Fresh weight (D)**	Genotype	1	91.05	0.000001	**Fresh weight (R)**	Genotype	1	610.92	0.000000
	Treatment	1	0.59	0.458391		Treatment	1	9.76	0.008777
	Genotype x Treatment	1	5.08	0.043763		Genotype x Treatment	1	0.29	0.600238
**Dry weight (D)**	Genotype	1	36.72	0.000057	**Dry weight (R)**	Genotype	1	38.63	0.000045
	Treatment	1	7.10	0.020645		Treatment	1	0.69	0.422220
	Genotype x Treatment	1	0.82	0.382027		Genotype x Treatment	1	0.02	0.882670
**Leaf area (D)**	Genotype	1	91.05	0.000001	**Leaf area (R)**	Genotype	1	140.67	0.000000
	Treatment	1	0.59	0.458391		Treatment	1	0.91	0.358278
	Genotype x Treatment	1	5.08	0.043763		Genotype x Treatment	1	0.29	0.600880

Supplementary Table S3. Content of soluble sugars in the leaves of WT and *flacca* tomato genotypes subjected to six-day water deficit (D) period followed by six-day period of re-watering (R). Respective controls corresponding to water-deficit condition (C_D) and re-watering (C_R) are presented. Values are presented as means ± SE (n = 4). Different letters denote significant differences between means according to Tukey HSD post hoc test $p \leq 0.05$. Small letters represent the differences in means of drought treatment, while caps letters represent re-watering treatment.

	WT		*Flacca*		WT		*flacca*	
	C_D	D	C_D	D	C_R	R	C_R	R
Hexose (µmol g^{-1} FW)								
Glucose	12.38 ± 0.95 ^{a}	12.60 ± 0.38 ^{a}	13.16 ± 0.73 ^{a}	19.94 ± 2.19 ^{b}	12.61 ± 0.83 ^{AB}	11.04 ± 0.30 ^{B}	17.76 ± 1.65 ^{C}	15.79 ± 0.46 ^{BC}
Fructose	8.65 ± 0.50 ^{a}	7.78 ± 0.93 ^{a}	7.83 ± 0.35 ^{a}	9.01 ± 0.91 ^{a}	8.35 ± 0.52 ^{A}	8.47 ± 0.19 ^{A}	11.70 ± 0.91 ^{AB}	15.27 ± 1.46 ^{B}
Galactose	0.25 ± 0.02 ^{a}	0.48 ± 0.09 ^{b}	1.38 ± 0.06 ^{c}	1.24 ± 0.18 ^{c}	0.26 ± 0.03 ^{A}	0.53 ± 0.11 ^{AB}	0.96 ± 0.08 ^{B}	1.61 ± 0.17 ^{C}
Pentose (µmol g^{-1} FW)								
Arabinose	0.15 ± 0.01 ^{a}	0.39 ± 0.06 ^{b}	0.79 ± 0.17 ^{c}	0.73 ± 0.07 ^{c}	0.08 ± 0.01 ^{A}	0.34 ± 0.08 ^{B}	0.78 ± 0.03 ^{C}	0.95 ± 0.09 ^{C}
Rhamnose	0.07 ± 0.01 ^{b}	0.08 ± 0.01 ^{a}	0.06 ± 0.01 ^{b}	0.08 ± 0.01 ^{b}	0.10 ± 0.01 ^{B}	0.07 ± 0.01 ^{A}	0.13 ± 0.01 ^{C}	0.13 ± 0.01 ^{C}
Xylose	0.49 ± 0.01 ^{a}	0.53 ± 0.04 ^{a}	0.69 ± 0.02 ^{b}	0.39 ± 0.07 ^{a}	0.50 ± 0.02 ^{A}	1.29 ± 0.08 ^{C}	0.69 ± 0.02 ^{AB}	0.81 ± 0.08 ^{B}
Disaccharides (µmol g^{-1} FW)								
Sucrose	0.69 ± 0.10 ^{bc}	0.41 ± 0.04 ^{a}	0.63 ± 0.04 ^{b}	0.78 ± 0.03 ^{c}	0.39 ± 0.06 ^{AB}	0.73 ± 0.06 ^{C}	0.32 ± 0.01 ^{A}	0.50 ± 0.04 ^{B}
Trehalose	0.08 ± 0.01 ^{a}	0.26 ± 0.04 ^{b}	0.02 ± 0.01 ^{a}	0.03 ± 0.01 ^{a}	0.13 ± 0.01 ^{B}	0.12 ± 0.02 ^{B}	0.03 ± 0.01 ^{A}	0.03 ± 0.01 ^{A}
Melibiose	0.24 ± 0.01 ^{b}	0.24 ± 0.02 ^{b}	0.11 ± 0.01 ^{a}	0.11 ± 0.01 ^{a}	0.23 ± 0.18 ^{B}	0.49 ± 0.01 ^{C}	0.14 ± 0.01 ^{A}	0.15 ± 0.01 ^{A}
Maltose	0.19 ± 0.02 ^{bc}	0.23 ± 0.02 ^{c}	0.13 ± 0.01 ^{a}	0.14 ± 0.01 ^{ab}	0.20 ± 0.02 ^{BC}	0.26 ± 0.04 ^{C}	0.11 ± 0.01 ^{A}	0.14 ± 0.01 ^{AB}
Oligosaccharides (nmol g^{-1} FW)								
Maltotriose	16.24 ± 2.22 ^{a}	21.46 ± 0.66 ^{ab}	21.81 ± 1.14 ^{ab}	24.48 ± 0.18 ^{b}	21.57 ± 1.06 ^{AB}	19.23 ± 0.76 ^{A}	23.23 ± 0.48 ^{B}	24.72 ± 1.16 ^{B}
Raffinose	43.28 ± 1.83 ^{b}	44.34 ± 1.91 ^{b}	31.03 ± 0.26 ^{a}	31.04 ± 0.39 ^{a}	47.38 ± 2.97 ^{BC}	54.91 ± 0.49 ^{C}	38.81 ± 0.54 ^{A}	39.69 ± 0.61 ^{AB}
Panose	13.44 ± 0.15 ^{a}	17.45 ± 3.44 ^{a}	12.44 ± 0.49 ^{a}	15.87 ± 1.44 ^{a}	18.32 ± 1.82 ^{A}	22.15 ± 0.56 ^{A}	40.87 ± 0.39 ^{B}	39.71 ± 1.27 ^{B}
Stachyose	12.33 ± 1.26 ^{ab}	12.98 ± 1.41 ^{b}	9.75 ± 0.43 ^{ab}	9.15 ± 0.27 ^{a}	16.29 ± 1.78 ^{AB}	19.27 ± 0.43 ^{B}	14.27 ± 0.59 ^{A}	14.93 ± 0.56 ^{AB}
Sugar alcohols (µmol g^{-1} FW)								
Sorbitol	1.03 ± 0.08 ^{a}	1.65 ± 0.13 ^{b}	4.16 ± 0.14 ^{c}	5.58 ± 0.71 ^{c}	1.69 ± 0.17 ^{A}	1.64 ± 0.20 ^{A}	5.87 ± 0.13 ^{B}	5.83 ± 0.58 ^{B}
**Total SS (µmol g^{-1} FW)**								
	24.31 ± 0.75 ^{a}	24.74 ± 1.04 ^{a}	29.04 ± 1.14 ^{a}	38.11 ± 3.97 ^{b}	24.64 ± 0.89 ^{A}	25.10 ± 0.64 ^{A}	38.61 ± 0.85 ^{B}	41.33 ± 1.72 ^{B}

Supplementary Table S4. Statistical analysis of the results (two-way ANOVA) to test the influence of the treatment (drought – D or re-watering – R) and two genotypes (WT and *flacca*) and their interactions on the soluble sugars content. Abbreviations: Glucose – Glc, Fructose – Fru, Galactose – Gal, Arabinose – Ara, Rhamnose – Rham, Xylose – Xyl, Sucrose – Suc, Trehalose – Tre, Maltose – Mal, Raffinose – Raf, Panose – Pan, Stachyose – Stach, Sorbitol – Sor.

Trait	Source of variation	df	F	*p*	Trait	Source of variation	df	F	*p*
**Glc (D)**	Genotype	1	17.17	0.000865	**Glc (R)**	Genotype	1	26.42	0.000245
	Treatment	1	11.85	0.003631		Treatment	1	3.38	0.090797
	Genotype x Treatment	1	10.54	0.005421		Genotype x Treatment	1	0.04	0.844564
**Fru (D)**	Genotype	1	0.084	0.777602	**Fru (R)**	Genotype	1	31.46	0.000115
	Treatment	1	0.04	0.831652		Treatment	1	4.17	0.063635
	Genotype x Treatment	1	2.0476	0.177969		Genotype x Treatment	1	3.64	0.080716
**Glc/Fru ratio (D)**	Genotype	1	17.023	0.001406	**Glc/Fru ratio (R)**	Genotype	1	2.83	0.118036
	Treatment	1	16.474	0.001585		Treatment	1	34.96	0.000071
	Genotype x Treatment	1	2.018	0.180921		Genotype x Treatment	1	5.81	0.032837
**Gal (D)**	Genotype	1	289.32	0.000000	**Gal (R)**	Genotype	1	67.08	0.000003
	Treatment	1	0.57	0.462625		Treatment	1	17.41	0.001293
	Genotype x Treatment	1	11.33	0.003927		Genotype x Treatment	1	3.03	0.107071
**Ara (D)**	Genotype	1	121.70	0.000000	**Ara (R)**	Genotype	1	185.79	0.000000
	Treatment	1	4.50	0.052168		Treatment	1	20.09	0.000377
	Genotype x Treatment	1	11.37	0.004555		Genotype x Treatment	1	0.86	0.368015
**Rham (D)**	Genotype	1	3.00	0.102478	**Rham (R)**	Genotype	1	122.33	0.000000
	Treatment	1	11.73	0.003477		Treatment	1	21.27	0.000599
	Genotype x Treatment	1	7.55	0.014311		Genotype x Treatment	1	17.73	0.001210
**Xyl (D)**	Genotype	1	0.78	0.390022	**Xyl (R)**	Genotype	1	5.34	0.039488
	Treatment	1	12.96	0.002402		Treatment	1	51.48	0.000011
	Genotype x Treatment	1	21.04	0.000304		Genotype x Treatment	1	27.76	0.000198
**Suc (D)**	Genotype	1	15.51	0.001176	**Suc (R)**	Genotype	1	9.23	0.010306
	Treatment	1	3.08	0.098583		Treatment	1	32.40	0.000100
	Genotype x Treatment	1	35.30	0.000021		Genotype x Treatment	1	2.21	0.162891
**Tre (D)**	Genotype	1	82.24	0.000001	**Tre (R)**	Genotype	1	255.73	0.000000
	Treatment	1	48.17	0.000016		Treatment	1	91.38	0.000001
	Genotype x Treatment	1	20.88	0.000644		Genotype x Treatment	1	0.34	0.567987
			15.85	0.001824				0.28	0.608257
**Mal (D)**	Genotype	1	27.49	0.000207	**Mal (R)**	Genotype	1	30.48	0.000132
	Treatment	1	1.94	0.189404		Treatment	1	4.22	0.062439
	Genotype x Treatment	1	0.33	0.575424		Genotype x Treatment	1	0.78	0.395179

**Raf (D)**	Genotype	1	46.39	0.000019	**Raf (R)**	Genotype	1	37.93	0.000049
	Treatment	1	4.93	0.046462		Treatment	1	3.86	0.073062
	Genotype x Treatment	1	2.87	0.116214		Genotype x Treatment	1	2.16	0.167528
**Pan (D)**	Genotype	1	0.66	0.431195	**Pan (R)**	Genotype	1	297.36	0.000000
	Treatment	1	2.79	0.120665		Treatment	1	1.32	0.273232
	Genotype x Treatment	1	0.11	0.747021		Genotype x Treatment	1	4.62	0.052707
**Stach (D)**	Genotype	1	15.51	0.001969	**Stach (R)**	Genotype	1	7.59	0.017412
	Treatment	1	0.03	0.866993		Treatment	1	2.37	0.149259
	Genotype x Treatment	1	0.15	0.702514		Genotype x Treatment	1	0.89	0.364404
**Sor (D)**	Genotype	1	192.00	0.000000	**Sor (R)**	Genotype	1	166.03	0.000000
	Treatment	1	15.54	0.001954		Treatment	1	0.08	0.782405
	Genotype x Treatment	1	1.22	0.291613		Genotype x Treatment	1	0.01	0.935445
**Total SS (D)**	Genotype	1	26.43	0.000245	**Total SS (R)**	Genotype	1	186.31	0.000000
	Treatment	1	7.05	0.021006		Treatment	1	2.04	0.179204
	Genotype x Treatment	1	5.88	0.032089		Genotype x Treatment	1	1.07	0.320500

Supplementary Table S5. Content of free amino acids (AAs) (µmol g^{-1} FW*)* in the leaves of WT and *flacca* tomato genotypes subjected to six-day water deficit (D) period followed by six-day period of re-watering (R). Respective controls corresponding to water-deficit condition (C_D) and re-watering (C_R) are presented. Values are presented as means ± SE (n = 4). Different letters denote significant differences between means according to Tukey HSD post hoc test $p \leq 0.05$. Small letters represent the differences in means of drought treatment, while caps letters represent re-watering treatment.

	WT		*flacca*		WT		*flacca*	
	C_D	D	C_D	D	C_R	R	C_R	R
Aspartate	1.48 ± 0.01 ^{a}	2.23 ± 0.09 ^{c}	2.75 ± 0.18 ^{b}	1.55 ± 0.06 ^{a}	1.32 ± 0.10 ^{A}	1.41 ± 0.20 ^{C}	3.58 ± 0.02 ^{A}	2.04 ± 0.07 ^{B}
Glutamate	1.44 ± 0.01 ^{a}	2.57 ± 0.15 ^{b}	2.61 ± 0.22 ^{b}	3.60 ± 0.34 ^{c}	1.37 ± 0.08 ^{A}	1.64 ± 0.12 ^{C}	4.43 ± 0.50 ^{AB}	2.20 ± 0.04 ^{B}
Glutamine	0.56 ± 0.01 ^{a}	0.86 ± 0.06 ^{b}	1.33 ± 0.09 ^{a}	2.37 ± 0.20 ^{c}	0.73 ± 0.01 ^{A}	0.83 ± 0.12 ^{B}	2.15 ± 0.24 ^{A}	1.01 ± 0.02 ^{A}
Serine	0.32 ± 0.01 ^{a}	0.56 ± 0.05 ^{b}	0.56 ± 0.05 ^{b}	0.77 ± 0.09 ^{c}	0.34 ± 0.03 ^{A}	0.27 ± 0.02 ^{C}	0.71 ± 0.05 ^{A}	0.53 ± 0.04 ^{B}
Alanine	0.32 ± 0.03 ^{a}	0.43 ± 0.01 ^{ab}	0.55 ± 0.08 ^{bc}	0.67 ± 0.11 ^{c}	0.62 ± 0.04 ^{B}	0.34 ± 0.01 ^{A}	0.68 ± 0.05 ^{B}	0.39 ± 0.01 ^{A}
Proline	0.27 ± 0.03 ^{a}	3.70 ± 0.81 ^{a}	0.35 ± 0.02 ^{c}	3.12 ± 0.24 ^{b}	0.22 ± 0.03 ^{A}	0.26 ± 0.04 ^{A}	0.32 ± 0.04 ^{A}	1.03 ± 0.15 ^{B}
Threonine	0.26 ± 0.03 ^{a}	0.36 ± 0.03 ^{ab}	0.32 ± 0.04 ^{a}	0.48 ± 0.07 ^{b}	0.25 ± 0.01 ^{A}	0.26 ± 0.04 ^{A}	0.51 ± 0.05 ^{B}	0.27 ± 0.01 ^{A}
Arginine	0.21 ± 0.02 ^{a}	0.20 ± 0.01 ^{a}	0.32 ± 0.03 ^{ab}	0.28 ± 0.05 ^{b}	0.31 ± 0.02 ^{B}	0.15 ± 0.01 ^{A}	0.25 ± 0.04 ^{B}	0.30 ± 0.03 ^{B}
Glycine	0.16 ± 0.02 ^{b}	0.13 ± 0.02 ^{b}	0.18 ± 0.02 ^{ab}	0.10 ± 0.01 ^{a}	0.27 ± 0.01 ^{C}	0.18 ± 0.01 ^{B}	0.23 ± 0.01 ^{A}	0.18 ± 0.01 ^{A}
Lysine	0.13 ± 0.02 ^{a}	0.20 ± 0.02 ^{a}	0.31 ± 0.03 ^{b}	0.39 ± 0.02 ^{c}	0.22 ± 0.09 ^{A}	0.19 ± 0.06 ^{A}	0.42 ± 0.14 ^{B}	2.88 ± 0.87 ^{C}
Leucine	0.12 ± 0.01 ^{ab}	0.15 ± 0.06 ^{bc}	0.19 ± 0.03 ^{c}	0.10 ± 0.01 ^{a}	0.22 ± 0.04 ^{A}	0.16 ± 0.06 ^{A}	0.25 ± 0.01 ^{A}	0.26 ± 0.07 ^{A}
Tyrosine	0.12 ± 0.01 ^{a}	0.21 ± 0.06 ^{b}	0.29 ± 0.01 ^{b}	0.56 ± 0.02 ^{c}	0.23 ± 0.02 ^{A}	0.24 ± 0.02 ^{A}	0.55 ± 0.17 ^{B}	0.16 ± 0.01 ^{A}
Phenylalanine	0.09 ± 0.01 ^{a}	0.12 ± 0.01 ^{a}	0.22 ± 0.01 ^{c}	0.15 ± 0.01 ^{b}	0.12 ± 0.01 ^{A}	0.10 ± 0.01 ^{A}	0.15 ± 0.02 ^{B}	0.17 ± 0.01 ^{B}
Asparagine	0.07 ± 0.01 ^{a}	0.12 ± 0.01 ^{b}	0.16 ± 0.01 ^{b}	0.33 ± 0.03 ^{c}	0.10 ± 0.01 ^{A}	0.12 ± 0.01 ^{B}	0.37 ± 0.04 ^{A}	0.14 ± 0.01 ^{A}
Valine	0.05 ± 0.01 ^{a}	0.09 ± 0.01 ^{b}	0.10 ± 0.01 ^{b}	0.17 ± 0.02 ^{c}	0.09 ± 0.01 ^{A}	0.06 ± 0.01 ^{A}	0.14 ± 0.02 ^{A}	0.08 ± 0.01 ^{B}
Isoleucine	0.05 ± 0.01 ^{b}	0.02 ± 0.01 ^{a}	0.05 ± 0.01 ^{b}	0.10 ± 0.01 ^{c}	0.10 ± 0.01 ^{A}	0.08 ± 0.02 ^{A}	0.06 ± 0.01 ^{A}	0.08 ± 0.01 ^{A}
Ornithine	0.02 ± 0.01 ^{a}	0.03 ± 0.01 ^{b}	0.03 ± 0.01 ^{b}	0.04 ± 0.01 ^{c}	0.02 ± 0.01 ^{A}	0.02 ± 0.01 ^{A}	0.03 ± 0.01 ^{A}	0.03 ± 0.01 ^{A}
**Total AA**	5.64 ± 0.11 ^{a}	11.98 ± 0.68 ^{b}	10.32 ± 0.71 ^{b}	14.76 ± 0.65 ^{c}	6.45 ± 0.17 ^{A}	6.31 ± 0.38 ^{A}	14.83 ± 1.21 ^{C}	11.75 ± 0.75 ^{B}

Supplementary Table S6. Statistical analysis of the results (two-way ANOVA) to test the influence of the treatment (drought – D or re-watering – R) and two genotypes (WT and *flacca*) and their interactions on the free amino acids content. Abbreviations: Aspartate – Asp, Glutamate – Glu, Glutamine – Gln, Serine – Ser, Alanine – Ala, Proline – Pro, Treonine – Tre, Arginine – Arg, Glycine – Gly, Lysine – Lys, Leucine – Leu, Tyrosine – Tyr, Phenilalanine – Phe, Asparagine – Asp, Valine – Val, Isoleucine – Ile, Ornithine – Orn.

Trait	Source of variation	df	F	*p*	Trait	Source of variation	df	F	*p*
**Asp (D)**	Genotype	1	10.63	0.006818	**Asp (R)**	Genotype	1	304.51	0.000000
	Treatment	1	6.15	0.029009		Treatment	1	76.49	0.000001
	Genotype x Treatment	1	115.92	0.000000		Genotype x Treatment	1	96.18	0.000000
**Glu (D)**	Genotype	1	50.71	0.000012	**Glu (R)**	Genotype	1	97.78	0.000000
	Treatment	1	46.58	0.000018		Treatment	1	28.75	0.000170
	Genotype x Treatment	1	0.22	0.646210		Genotype x Treatment	1	46.85	0.000018
**Gln (D)**	Genotype	1	198.30	0.000000	**Gln (R)**	Genotype	1	69.92	0.000002
	Treatment	1	67.37	0.000003		Treatment	1	29.52	0.000152
	Genotype x Treatment	1	20.48	0.000695		Genotype x Treatment	1	42.42	0.000029
**Ser(D)**	Genotype	1	29.07	0.000162	**Ser (R)**	Genotype	1	151.51	0.000000
	Treatment	1	30.05	0.000140		Treatment	1	25.64	0.000278
	Genotype x Treatment	1	0.16	0.694734		Genotype x Treatment	1	4.55	0.054161
**Ala (D)**	Genotype	1	22.38	0.000488	**Ala (R)**	Genotype	1	5.57	0.036084
	Treatment	1	5.48	0.037267		Treatment	1	141.91	0.000000
	Genotype x Treatment	1	0.00	0.964919		Genotype x Treatment	1	0.00	0.970112
**Pro (D)**	Genotype	1	7.39	0.018665	**Pro (R)**	Genotype	1	61.14	0.000005
	Treatment	1	1803.36	0.000000		Treatment	1	44.81	0.000022
	Genotype x Treatment	1	14.67	0.002392		Genotype x Treatment	1	36.85	0.000056
**Tre(D)**	Genotype	1	6.18	0.028614	**Tre (R)**	Genotype	1	43.77	0.000025
	Treatment	1	14.79	0.002329		Treatment	1	29.42	0.000154
	Genotype x Treatment	1	0.73	0.409787		Genotype x Treatment	1	33.44	0.000087
**Arg (D)**	Genotype	1	16.95	0.001427	**Arg (R)**	Genotype	1	5.47	0.037413
	Treatment	1	1.62	0.227618		Treatment	1	6.44	0.026071
	Genotype x Treatment	1	0.36	0.560661		Genotype x Treatment	1	24.15	0.000357
**Gly (D)**	Genotype	1	0.04	0.842515	**Gly (R)**	Genotype	1	11.53	0.005316
	Treatment	1	18.49	0.001032		Treatment	1	175.49	0.000000
	Genotype x Treatment	1	4.63	0.052375		Genotype x Treatment	1	16.39	0.001613
**Lys (D)**	Genotype	1	105.22	0.000000	**Lys (R)**	Genotype	1	11.37	0.005547
	Treatment	1	18.19	0.001098		Treatment	1	1.9724	0.185546
	Genotype x Treatment	1	0.13	0.727969		Genotype x Treatment	1	2.1866	0.164981

**Leu (D)**	Genotype	1	0.75	0.403964	**Leu (R)**	Genotype	1	0.75	0.403964
	Treatment	1	4.75	0.049925		Treatment	1	4.75	0.049925
	Genotype x Treatment	1	34.90	0.000072		Genotype x Treatment	1	34.90	0.000072
**Tyr (D)**	Genotype	1	155.71	0.000000	**Tyr (R)**	Genotype	1	3.57	0.083094
	Treatment	1	73.46	0.000002		Treatment	1	9.49	0.009518
	Genotype x Treatment	1	17.89	0.001168		Genotype x Treatment	1	10.83	0.006445
**Phe (D)**	Genotype	1	109.74	0.000000	**Phe (R)**	Genotype	1	48.31	0.000015
	Treatment	1	6.81	0.022854		Treatment	1	0.38	0.550781
	Genotype x Treatment	1	38.40	0.000046		Genotype x Treatment	1	7.20	0.019917
**Asn (D)**	Genotype	1	154.02	0.000000	**Asn (R)**	Genotype	1	85.79	0.000001
	Treatment	1	93.75	0.000001		Treatment	1	41.27	0.000033
	Genotype x Treatment	1	25.79	0.000271		Genotype x Treatment	1	62.77	0.000004
**Val (D)**	Genotype	1	53.35	0.000009	**Val (R)**	Genotype	1	22.46	0.000481
	Treatment	1	40.17	0.000037		Treatment	1	27.58	0.000204
	Genotype x Treatment	1	2.44	0.144336		Genotype x Treatment	1	5.78	0.033235
**Ile (D)**	Genotype	1	372.71	0.000000	**Ole (R)**	Genotype	1	3.27	0.095841
	Treatment	1	31.64	0.000112		Treatment	1	0.01	0.908896
	Genotype x Treatment	1	245.33	0.000000		Genotype x Treatment	1	4.27	0.061041
**Orn (D)**	Genotype	1	34.28	0.000078	**Orn (R)**	Genotype	1	8.95	0.011233
	Treatment	1	22.21	0.000503		Treatment	1	0.27	0.615684
	Genotype x Treatment	1	0.25	0.626623		Genotype x Treatment	1	0.53	0.481160
**Gly/Ser ratio (D)**	Genotype	1	14.525	0.005154	**Gly/Ser ratio (R)**	Genotype	1	172.77	0.000001
	Treatment	1	44.578	0.000156		Treatment	1	4.12	0.076853
	Genotype x Treatment	1	0.902	0.370111		Genotype x Treatment	1	8.05	0.021928
**Total AA (D)**	Genotype	1	39.68	0.000233	**Total AA (R)**	Genotype	1	55.94	0.000710
	Treatment	1	83.05	0.000017		Treatment	1	5.87	0.041625
	Genotype x Treatment	1	2.58	0.146667		Genotype x Treatment	1	4.83	0.059093
